# Collaborative and Cooperative Hospital “In-House” Medical Device Development and Implementation in the AI Age: The European Responsible AI Development (EURAID) Framework Compatible With European Values

**DOI:** 10.2196/80754

**Published:** 2026-01-29

**Authors:** Anett Schönfelder, Maria Eberlein-Gonska, Manfred Hülsken-Giesler, Florian Jovy-Klein, Jakob Nikolas Kather, Elisabeth Kohoutek, Thomas Lennefer, Elisabeth Liebert, Myriam Lipprandt, Rebecca Mathias, Hannah Sophie Muti, Julius Obergassel, Thomas Reibel, Ulrike Rösler, Moritz Schneider, Larissa Schlicht, Hannes Schlieter, Malte L Schmieding, Nils Schweingruber, Martin Sedlmayr, Reinhard Strametz, Barbara Susec, Magdalena Katharina Wekenborg, Eva Weicken, Katharina Weitz, Anke Diehl, Stephen Gilbert

**Affiliations:** 1 Else Kroener Fresenius Center for Digital Health Faculty of Medicine and University Hospital Carl Gustav Carus TUD Dresden University of Technology Dresden, Saxony Germany; 2 University Hospital Carl Gustav Carus Dresden Germany; 3 Department of Nursing Science Institute of Health Research and Education University of Osnabrück Osnabrück Germany; 4 Institute for Technology and Innovation Management (TIM) RWTH Aachen University Aachen Germany; 5 Department of Medicine I Faculty of Medicine and University Hospital Carl Gustav Carus TUD Dresden University of Technology Dresden, Saxony Germany; 6 Medical Oncology, National Center for Tumor Diseases (NCT) University Hospital Heidelberg Heidelberg Germany; 7 Pathology & Data Analytics Leeds Institute of Medical Research at St James's University of Leeds Leeds United Kingdom; 8 Luther Rechtsanwaltsgesellschaft mbH Cologne Germany; 9 Department for Prevention AOK Federal Association Berlin Germany; 10 Department for Digital Transformation University Medicine Essen Essen Germany; 11 Institute of Medical Informatics University Hospital RWTH Aachen Aachen, North Rhine-Westphalia Germany; 12 Department for Visceral, Thoracic and Vascular Surgery Faculty of Medicine and University Hospital Carl Gustav Carus TUD Dresden University of Technology Dresden Germany; 13 University Medical Center Hamburg - Eppendorf Hamburg Germany; 14 Federal Institute for Occupational Safety and Health Berlin Germany; 15 Institute for Occupational Safety and Health of the German Social Accident Insurance Sankt Augustin Germany; 16 Faculty of Humanities and Social Sciences Karlsruhe Institute of Technology Karlsruhe Germany; 17 Faculty of Business and Economics TUD Dresden University of Technology Dresden Germany; 18 German Federal Ministry of Health Berlin Germany; 19 Institute for Medical Informatics and Biometry Faculty of Medicine and University Hospital Carl Gustav Carus TUD Dresden University of Technology Dresden Germany; 20 Wiesbaden Institute for Healthcare Economics and Patient Safety (WiHelP) Wiesbaden Germany; 21 ver.di Berlin Germany; 22 Department of Artificial Intelligence Fraunhofer Heinrich Hertz Institute Berlin Germany

**Keywords:** AI Act, digital transformation, in-house medical device development, agentic AI, AI life cycle, artificial intelligence

## Abstract

The last years have seen an acceleration in the development and uptake of artificial intelligence (AI) systems by “early adopter” hospitals, caught between the pressures to “perform” and “transform” in a struggling health care system. This transformation has raised concerns among health care providers as their voices and location-specific workflows have often been overlooked, resulting in technologies that fail to integrate meaningfully into routine care and worsen rather than improve care processes. How can positive AI implementation be carried out in health care, aligned with European values? Based on a perspective that spans all stakeholders, we have created EURAID (European Responsible AI Development), a practical, human-centric framework for AI development and implementation based on agreed goals and values. We illustrate this approach through the co-development of a narrow-purpose “in-house” AI system, designed to help bridge the AI implementation gap in real-world clinical settings. This example is then expanded to address the broader challenges associated with complex, multiagent AI systems. By portraying all key stakeholders across the AI development life cycle and highlighting their roles and contributions within the process, real use cases, and methods for achieving iterative consensus, we offer a unique practical approach for safe and fast progress in hospital digital transformation in the AI age.

## The Transformation of Future Medicine Through Artificial Intelligence Technologies

Will the slogans already heard in health care system strikes, such as “Trust Nurses, Not AI” and “AI has got to go!”[1,2], become more common? They reflect growing concerns about the evolving role of health care professionals (HCPs) in a changing health system, which persist despite reports that 20% of National Health Service (NHS) doctors are already using artificial intelligence (AI) daily [3]. Although the importance of digital transformation to enhance the efficiency of care delivery and to provide better models of care suited to modern age [4-6] is well recognized within care systems [7-11], it often cannot be comprehensively addressed, as health care systems worldwide find themselves caught between the need to both “perform” and “transform” in a system facing “firefighting” ongoing challenges [12-17]. The application of AI technologies has the potential to address some of those aspects (Table 1), as it can speed digital transformation and can (at least if applied well and if the associated potential barriers and uncertainties are jointly recognized and resolved) make health care more accessible, effective, and economically sustainable [18]. Examples of the positive impact of good AI implementation are (1) enhancement of clinical practice, particularly in areas such as diagnosis and personalized medicine [11,19,20]; (2) workflow improvements, by supporting administrative tasks such as transcription, patient communication, and patient-related recordkeeping [21,22]; and (3) increased operational efficiency, through the optimization of routine processes, enabling HCPs to work in a more patient-centered way [23], and potentially contribute to cost reductions [24,25]. With the recent introduction of “agentic AI” [26-29] and autonomous AI-enabled systems [30,31], far more systematic complexity can be handled by AI [32].

**Table 1:** Problems artificial intelligence (AI)–enabled transformation can address, approaches, challenges, and possible unintended consequences.

Current health system problems	Possible digital and AI^a^ solutions	Implementation challenges and risks
Administrative workload unrelated to direct patient care [33,34], inefficient workflows, and fragmented communication burden on HCPs^b^.	Automation of administrative and routine tasks, and AI-driven workflow optimization, allowing people to focus on patients.	Different perspectives on which tasks to automate. Increase in workload in some cases. Risk of overreliance on AI outcomes with insufficient human oversight. Automation of the current way of providing care without restructuring and rethinking processes. Concerns about job security, the transformation of job roles, and medical malpractice.
Stress, duplication (eg, medical history) [35], and discontinuous care resulting from disconnected devices, limited interoperability, and manual coordination.	Adjusting the hospital’s IT environment as an AI-sustained platform, characterized by high interoperability in itself and with other providers supporting seamless patient journeys.	Deficient data quality, data silos, inadequate computational resources, a shortage of specialized expertise, and poor or nonexistent infrastructure between providers. Concerns about safety and regulation.
Poor information flow and HCP training deficit.	AI-supported knowledge management to build confidence in usage.	Shortage of HCPs limits time for training. Various adoption readiness levels among HCPs. Concerns about trust in technologies.

^a^AI: artificial intelligence.

^b^HCP: health care professional.

However, AI is not a panacea, and initial evaluations of real-world performance in clinical settings are mixed [36-38]. One reason is that AI implementation projects have often underestimated the importance of individual AI medical devices operating as interconnected clinical and technological infrastructures rather than being a collection of isolated, standalone algorithms. AI in health care over the next years needs to be seen as interacting, interdependent, and flexible applications [39], involving both broad- and narrow-purpose tools and models that closely interact with and reshape human workflows, while simultaneously, human workflows, adaptations, and experience reshape the use of AI, particular to the local setting and local approach to health care delivery.

## Integration of Interactive AI Systems in Clinical Workflows Requires HCPs at the Core, Not as Observers

This future model needs HCPs at its core, not only as users interacting with AI systems, but as active participants in their co-design, procurement, implementation, monitoring, and evaluation. This idea is rooted in organizational and implementation theories, such as the “socio-technical systems theory” [40], that emphasizes the importance of a holistic perspective to jointly bridge human and technological capabilities, particularly in the context of autonomous technologies [41,42], and the “normalization process theory” [43], which acknowledges users’ cognitive participation and collective action as key determinants in implementing, embedding, and integrating complex and new interventions (eg, AI systems) in daily practice [44,45]. “Human-centered AI” can take a cross-theoretical perspective by viewing AI systems not as stand-alone technologies, but as integral components of a broader sociotechnical system. Two perspectives are relevant: humans being able to understand AI and AI being able to understand humans [46]. For example, explainable AI (XAI) methods should not only address the technical transparency of machine learning models but also focus on human understanding [47]. On the other hand, AI systems need to take into account the needs, requirements, and mental models of humans [48] and the context of clinical decisions [49] to create explanations that are supportive in the clinical setting.

**Figure 1:**
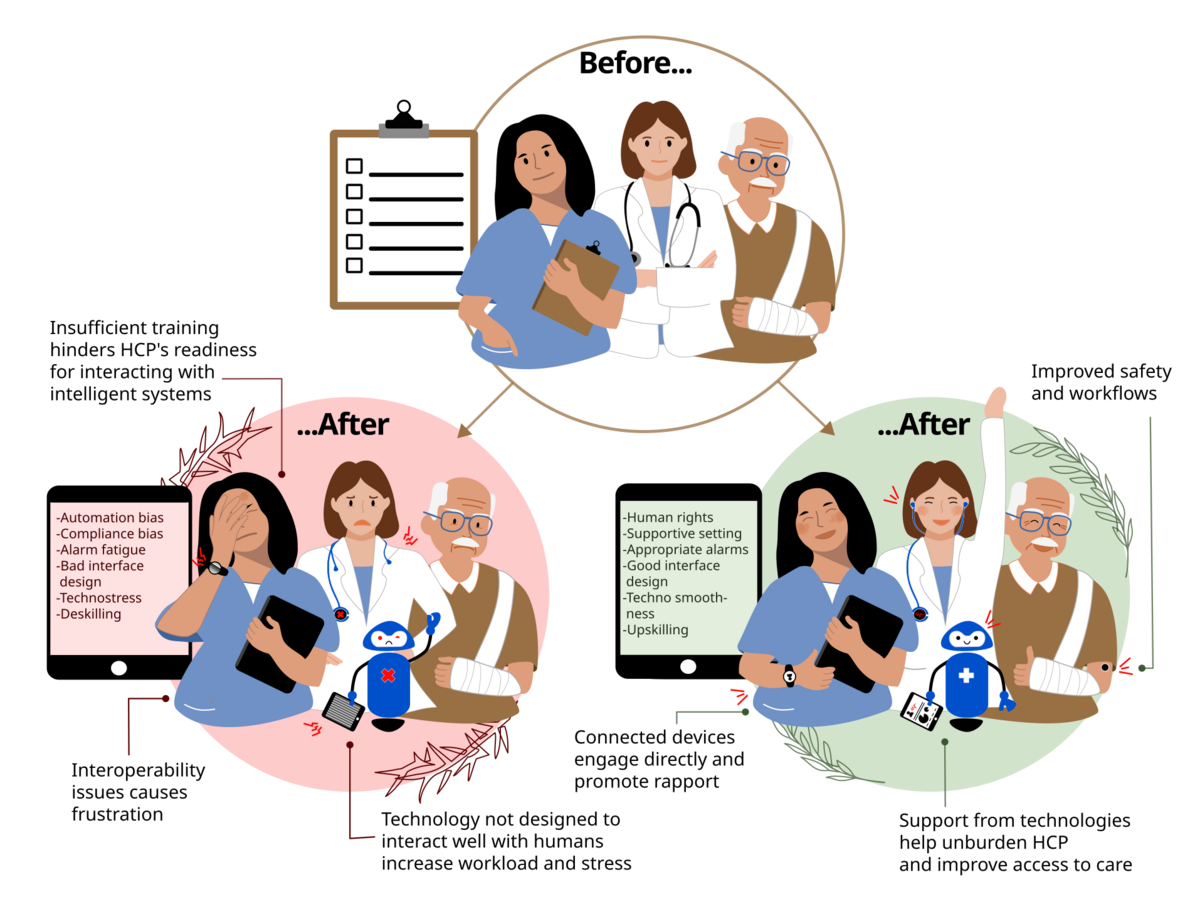
The introduction of artificial intelligence (AI) into clinical workflows is changing everyday clinical care and could, at least theoretically, enhance satisfaction, empower, upskill, and provide a better work environment and better interactions for health care professionals (HCPs) and patients; however, the reality is often much less positive. The upper circle is showing the current situation of health care delivery, which is characterized by a low level of digitalization and an ever-increasing amount of nonpatient-related activities, causing moderate satisfaction and happiness among both HCPs and patients. Care delivery transformation through AI can bring positive effects as shown in the green circle on the right (such as delivering better, more efficient and even more patient-centered care through optimized processes and well-balanced support systems) or, as is frequently the case, negative effects (red circle on the left), causing frustration, disconnection and stress of HCPs and patients because of interoperability issues with AI implementations that were never properly designed with the user needs in mind.

Yet, despite the substantial body of research on theoretical foundations, the translation of the underlying principles into everyday implementation of AI systems and clinical reality is lagging behind [50-54], often key aspects are neglected, and many implementation projects fail [55]. Problems often begin during the development of AI systems, which are frequently designed and tested in settings that are far removed from the everyday realities of clinical practice [56], and with HCPs and location-specific workflows often overlooked. The consequences of systems designed without sustained input from HCP and patients [57] are visible as they fail to demonstrate their suitability and worsen rather than improve processes, leading to the perception that the introduction of digital technologies into health care adds to the burden [57,58] (Figure 1), although general relief through well-implemented work aids would be very welcomed. That misalignment has been associated with increased stress among HCPs [59] (including “technostress” [60,61]), disconnected patient care [62,63], and has even resulted in other unintended negative consequences, such as HCPs resisting the use of the technologies [15], using technologies in unanticipated ways [64], or developing workarounds that may endanger patient care [65]. Insufficient digital health literacy and training among HCPs amplify these effects, leaving HCPs unprepared for the demands of interacting with intelligent systems [66]. Other consequences appearing in real-world implementation are model uncertainty [67], “AI hallucinations” or clinically harmful recommendations, bias [14], and context misalignment [68], which risk fragmented care and diminish patients’ trust in technology-assisted decisions.

## Improving Adoption by Co-Development Across the AI Life Cycle

### Overview

The real-world challenges discussed underscore that successful AI development and implementation are less a technical task than a comprehensive change management process [57] that needs active participation, transparent governance, continuous feedback, and development beyond technical metrics, including systematic real-world evaluation of human-AI interaction, and a focus on non-technical design criteria such as usability, workflow fit, trust, and acceptance.

To bridge this gap, we propose EURAID (European Responsible AI Development), a practical framework of human-centric AI development and implementation in hospitals, which is cooperative and collaborative and based on shared goals in accordance with European values according to Article 2 of the Treaty on European Union (TEU; ie, human dignity [69,70], freedom [69,71], democracy [72], equality [69], rule of law [73], and human rights [69,74]) and European laws (Table 2).

**Table 2:** Regulations in the European Union and its member states that guide AI use in health care (nonexhaustive).

Regulation or law	Scope	Approach
Medical Device Regulation (MDR; 2017/745)	Governs medical devices (including digital systems) used for diagnostic or therapeutic purposes.	The medical devices’ intended purpose defines the associated performance claims, which must be substantiated through clinical evaluation. GSPRs^a^ must be met, including structured risk management (ISO^b^ 14971:2019), a certified QMS^c^ (ISO 13485:2016), usability engineering (IEC^d^ 62366-1:2015+A1:2020), and a planned and documented development process (IEC 62304:2006+A1:2015), depending on the respective product category.
Artificial Intelligence Act (AI Act; 2024/1689)	Governs the development, market entry, and use of AI^e^ systems.	Classifies high-risk AI systems (including AI-enabled medical devices, class IIa+) and GPAI^f^ (that can perform a wide range of tasks, not limited to one clear intended purpose) and LLM^g^ models, depending on both the function performed and the systems’ intended purpose. Additional transparency obligations apply for certain systems such as emotion recognition, biometric categorization, and interactive or generative AI.
EU Occupational Safety and Health Directive (89/391/EEC 1989) and national laws	Ensures workers’ health and safety.	Systematic risk assessments and preventive measures. Worker consultation and participation.
Professional regulations (eg, Federal Medical Code for doctors) and labor laws (eg, German Works Constitution Acts)	Defines autonomy and participation rights of HCPs^h^.	Protection of professional independence in decision-making. Co-determination rights of employee representatives, for example, when adopting systems that monitor behavior and/or performance.

^a^GSPR: general safety and performance requirements.

^b^ISO: International Organization for Standardization.

^c^QMS: quality management system.

^d^IEC: International Electrotechnical Commission.

^e^AI: artificial intelligence.

^f^GPAI: general-purpose artificial intelligence.

^g^LLM: large language model.

^h^HCP: health care professional.

In detail, we describe the appropriate stakeholder circle, the approaches needed for implementing new and highly integrated, localized, and adaptive AI models, and optimal techniques for building consent. While this paper emphasizes that AI systems are increasingly evolving into system-level tools with broad intended purposes, it is nevertheless valuable to explore the development of a narrow-purpose, limited-functionality tool as a simple entry point in the consideration of AI system implementation. This example serves as a foundation for discussing the broader challenges associated with a broad intended purpose and multiagent AI systems. We describe the co-development of an “in-house” AI system [75] that is developed within a health institution to address specific needs [76,77], rather than the implementation of an externally developed “off the shelf” AI system, as this allows more aspects of the collaborative process to be described.

This pragmatic approach was developed in part through in-depth individual consultation and 4 flexible multistakeholder workshops, which are described in more detail in Table 3. By bringing together all the relevant players in the health care ecosystem, we were able to set agreed goals and processes for the development, integration, use, and oversight of health AI. These insights from the workshops informed aspects of the development of the overall framework presented in this viewpoint, alongside the perspective of the authors.

**Table 3:** Methodological design of the stakeholder workshops. Since workshops are platforms to jointly identify and explore complex domains, and help to gain relevant insights beyond the individual stakeholders’ scope of knowledge [78], they offer a valuable basis for a framework that has consensus-building at its core.

Aspect	Approach
Stakeholder definition	An individual or group who is affected by or can influence the digital transformation in hospitals, particularly with a focus on AIa-enabled systems.
Identification of stakeholders	Stakeholders were identified using the 7Ps framework [79], which serves as a guide for engaging diverse and relevant interest groups. We modified the categories and definitions of the 7Ps according to our context: Patients and the public: As this is not a traditional patient-focused study, but rather a practical, expert-driven implementation guide for human-centric digital transformation in a hospital setting, stakeholders were viewed both as domain experts and as potential patients. Additionally, we had feedback from two different international patient representative organizations. Providers: Individuals who provide care to patients and offer relevant insights from their clinical work were included. The selected clinicians represent various medical fields, including psychology, and are balanced in their seniority and professional position. Purchasers: Since digital transformation must be financed individually by each hospital, we included stakeholders responsible for the high-level management of digital transformation in large hospitals who manage strategic decisions about cost underwriting based on a specific internal budget. Payers: In Germany, digital hospital transformation is supported through federal programs. Therefore, we involved stakeholders working at the Federal Ministry of Health and stakeholders who are actively translating those programs into clinical practice. Additionally, we included employees of insurance companies, as insurers play, in general, a critical role in creating patient-centric digital ecosystems and in incentivizing digital health solutions. Policy makers: Policy makers and supporters of digital transformation in hospitals were included, particularly those who support a human-centric approach while ensuring the rights of HCPs^b^ and patients are in place, spanning stakeholders from labor unions to occupational health and safety experts, as well as relevant legal and ethical perspectives. Product makers: As EURAID^c^ highlights the need for a well-balanced stakeholder group developing and implementing AI in health care, the stakeholders representing the “in-house” manufacturers are in their profession AI system developers, psychologists and human-centered AI development professionals, as well as experts in medical device regulation, quality and clinical risk management, medical informatics, and in occupational health and safety at work. Principal investigators: The researchers included were from a background of clinical AI, medical device regulation, nursing science, medical informatics, digital health, patient safety, psychology, and ethics.
Stakeholder engagement	Objectives: The goal of stakeholder engagement was to achieve a common agreement on the theme by balancing the differences of individual viewpoints (eg, between calls for greater space for innovation or rather tighter regulation), and developing a framework that all stakeholders agree with. Methods: Stakeholders were engaged through participatory workshops (three dealt with relevant aspects EURAID should focus on and were initiated by the German Federal Institute for Occupational Safety and Health (BAuA), in 2024 and 2025, with 25, 24, and 17 participants respectively; and one dealt with aspects of HCP integration and current health system problems AI-enabled transformation might solve (Table 1) and was organized by the Else Kröner Fresenius Center for Digital Health in February 2025, with 5 participants). The participating stakeholders spanned the whole 7P categories. Based on this data and a critical review of the literature exploring existing frameworks and gaps, AS and SG developed the concept for the paper and wrote the first draft of EURAID. The stakeholders reviewed the paper, validated its content, and provided further expert insights during a 4-month iterative consensus process.

^a^AI: artificial intelligence.

^b^HCP: health care professional.

^c^EURAID: European Responsible AI Development.

### Step 1: Comprehensive and Inclusive Stakeholder Involvement to Build Consensus and Ensure Goal-Oriented Development and Implementation

The selection and active participation of stakeholders and the building of consensus are critical to the success of the AI system development and implementation. The stakeholders involved should be balanced in disciplinarity (clinical, technical, and administrative [80]) and operational responsibilities (professional positions, employee representatives, etc) as well as in age and gender. In Table 4, we highlight the key stakeholders involved, and in particular their role in the implementation process. Each stakeholder is selected for their contribution, ranging from strategic aspects (management board) to safety perspectives (employee representatives, quality management, clinical experts, and users), and data-driven issues (AI system developer, data scientists, and IT and regulatory specialists). In principle, stakeholders in their profession are not mutually exclusive; instead, one could fulfill several roles simultaneously.

**Table 4:** Key stakeholders and their roles in shaping and guiding AI development and implementation in health care. Each stakeholder is selected for their contribution to the process and expertise.

Stakeholder		Important areas of stakeholder involvement and key aspects they can address
Management Board 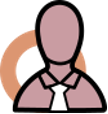		The management board sets an overall vision and strategy, leading change management [57,81], and providing investment [82] in staff, hardware, and supporting infrastructure [17]. They foster an institutional culture that tolerates experimentation (and failure) [80], serve as the institution’s most credible communicator (ensuring transparency around risks and benefits), and manage external relationships by forging alliances with industry innovators, researchers, professional associations, and policymakers.
Employee Representatives 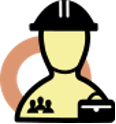		The foremost priority of employee representatives is to defend and improve working conditions, including occupational safety, workload management, and job security. Although large-scale staff redundancies are unlikely consequences of the near-term implementation of AI^a^ in hospital health care systems, which are operating against a backdrop of large staff shortage [83,84], anxiety about automation and transformation of job roles is real [85]. Employee representatives ensure that AI is implemented in a way that eases staff workload and safeguards their well-being and autonomy. In the mid- to long-term, they also negotiate fair compensation policies [86] and career development frameworks that reflect changing roles and skills in an increasingly digital workplace.
**AI-System Owner and team^b^** 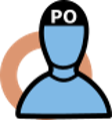		The AI system owner holds primary accountability for the system’s performance, safety, and operational impact. They lead the project and ensure alignment with strategic goals and regulatory compliance, while understanding the users’ “pain points” both from a clinical and organizational perspective. Their responsibilities include bridging the communication gap between technical and nontechnical language, balancing different perspectives, and developing educational approaches [66] to increase user adoption.
	Clinical Experts 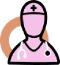	Clinical experts identify clinical relevance and utility, which are interpreted and transcribed into a specific scope (intended purpose that specifies clinical indication and initial target group). They provide crucial input to clinical validation and safety, ensuring the AI system integrates effectively into workflows, as well as initiate, oversee, and conduct clinical trial–based AI studies.
	AI System Developer 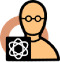	To design and develop machine learning algorithms tailored to specific needs, the AI system developer must integrate and harmonize data from different sources [15]. They also validate the AI model and detect and mitigate model bias to ensure the systems are fair, scalable, adaptable, and verifiable in real-world environments [87].
	Users (HCP^c^ or patient) 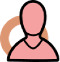	Users with varying levels of digital literacy [57,88] provide real-world, iterative feedback on the system’s usability, workflow integration, and perceived value. They often become multipliers for AI adoption, and by their active participation in co-designing educational materials [66], they support evolving digital competence among peers.
	Data Scientist 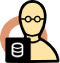	The data scientist safeguards the quality of the data foundation on which the AI system depends during preparation, collection, and checking of the data, for example, by keeping data collection protocols and detecting data imbalance, bias, or outliers across age, sex, gender, race, or ethnicity to prevent disparities and underperformance before they arise [89].
	IT Specialists 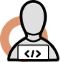	This role provides the essential technical infrastructure and ensures secure, seamless integration with existing systems, like EHR^d^ platforms or laboratory systems, requiring technical, syntactic, semantic, and organizational interoperability [15,90]. Beyond integration, they build and maintain structures for data security, access control, and real-time support, and establish data backup and disaster-recovery systems.
	Regulatory Specialists 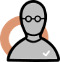	Regulatory Specialists provide expertise in medical device and AI law, data protection, and human rights. They ensure regulation standards (like the MDR^e^ and the AI Regulation) are met throughout the product lifecycle, which is essential to mitigate legal risks and prevent potential breaches.
	Notified Body 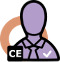	The role of the Notified Body is to assess whether medical devices meet European legislation, like MDR. This includes determining the correct classification, evaluating legal compliance, and reviewing technical documentation [75,91]. The Notified Body only has a direct role where a CE^f^-mark is sought for medium or high-risk AI systems.
Quality Management 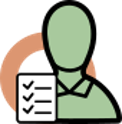		Quality management ensures continuous patient safety by monitoring and measuring performance, outcomes, and the integrity of clinical workflows [87]. They establish comprehensive risk management systems (eg, handling device failures or malfunctions) [87] and drive standardization. This role also promotes safe system use by co-designing educational programs [66] for both HCP and patients.

^a^AI: artificial intelligence.

^b^Role of the stakeholders whose input is coordinated through the AI-System Owner.

^c^HCP: health care professional.

^d^EHR: electronic health record.

^e^MDR: Medical Device Regulation (2017/745).

^f^CE: Conformité Européenne.

An interactive environment, with all critical stakeholder groups adequately represented, enables and encourages the integration of stakeholder insights and experiential learnings, while promoting careful consideration of how AI systems are best built to be suited to clinical workflows, as well as where existing workflows may need to be modified to adapt to the AI system. This does not mean that every stakeholder group is involved in every decision and has an equal say in the progress of digitalization. Creating this impression could lead to disillusionment and eroded trust in digitalization, and would probably slow down the whole process. Each stakeholder group is involved in some part of the process, with their precise stages of involvement and roles depending on their potential contribution to the process, and it is essential that each stakeholder is aware of the degree of their involvement.

**Figure 2:**
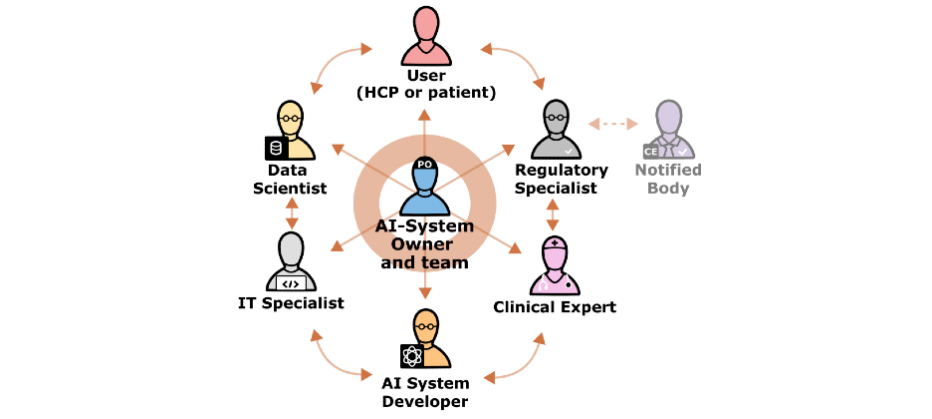
The (ongoing) product development in a dynamic team led by the AI-System Owner. The AI-System Owner fulfils a crucial role as he is leading a core team of relevant stakeholders during the process of development and implementation. In a hospital setting, team members will often fulfill several roles simultaneously. AI: artificial intelligence; HCP: health care professional.

A crucial success factor alongside the development and implementation is the role of the “product owner,” who takes the coordinating lead. As in-house development in health care institutions often does not have a commercial development focus, we use the term “AI-System Owner” to denote the “product owner.” Although the title may vary by organization, this role usually combines both the entire lifecycle product ownership responsibilities and the domain expertise in health care and AI. The absence of a single person taking responsibility for the development and performance of the system will generally result in a range of negative consequences, such as poor stakeholder communication, a lack of clear vision, scope, and prioritization, and other issues, as real-world examples [92] have shown. We therefore highlight the AI-System Owner as a central stakeholder leading a team of other stakeholders (Figure 2).

### Step 2: Agreement on the Overall Goals and “Device” Purpose

The collaborative and effective implementation of an AI system into clinical workflows starts with a collective agreement on the goals of the implementation, for example, using methods such as SMART (specific, measurable, attainable, relevant, and time-bound), particularly the specific user (generally an HCP or patient) whose needs the system is intended to address. These identified needs are then interpreted and transcribed into a specific scope of the device, known as the “Intended Purpose,” which specifies the clinical indication, how the system addresses this clinical indication, and the (initial) target group needs.

**Figure 3:**
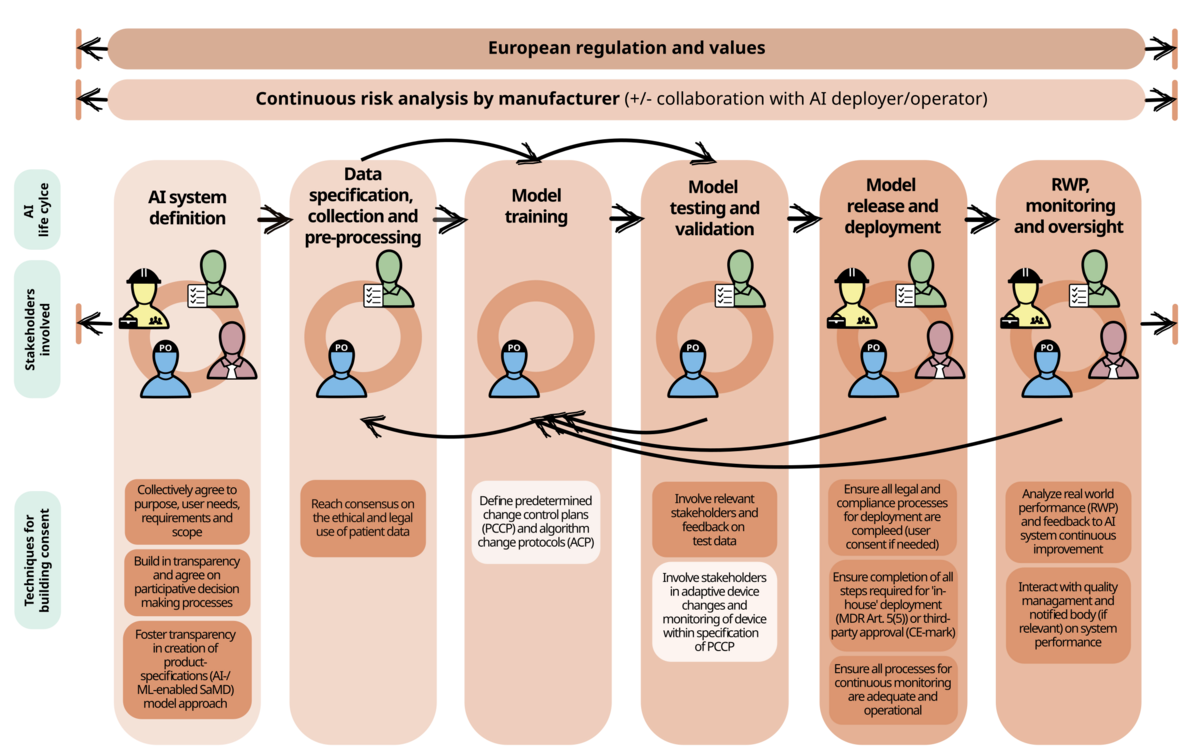
Stakeholder interaction and consent-building across the AI life cycle. The figure describes the co-development of an “in-house” AI system, ie, one that is developed within a health care organization to address specific needs. During the development phases, which build on each other and can be repeated, different groups of stakeholders interact to improve the AI system by providing feedback and optimizing the system’s adaptation to health care professional workflows. Building consent with a range of different stakeholders with varying levels of experience and backgrounds is not easy. We therefore highlight techniques for building consent at each stage of development to ensure an efficient and safe process that is in line with European values and regulations. AI: artificial intelligence; MDR: Medical Device Regulation; ML: machine learning; PCCP: predetermined change control plan; RWP: real-world performance; SaMD: software as a medical device.

Although the regulations for AI-system design and implementation do not formally require the direct involvement of any other health care system actors than the “user” of the AI and its “deployer” (in a broad sense), we argue that the sustainable and beneficial implementation of AI systems needs early and proportional agreement on goals and input from all stakeholders. This includes discussion between the management board, employee representatives, quality management, and the AI-System Owner and their team (Figure 3). Later product development steps require feedback between the AI-System Owner team (including clinical experts and the users of the system), and selected stakeholders (as shown in Figure 3), with management “checkpoints” periodically to ensure that the development of the AI-system is following the initially agreed plan for the AI system. Given the complexity of multistakeholder involvement, it is useful to have a set of rules for working together at the beginning, and to repeatedly build consent along the AI development life cycle, for which we highlight techniques in Figure 3.

### Step 3: AI System Development “In-House”

While generally medical devices must undergo a conformity assessment procedure and must be marked with a CE (Conformité Européenne)-mark before being used, the European Union (EU) exempts certain devices from this general obligation and allows individual health institutions to develop and use “in-house” medical AI systems involved in the diagnosis or therapy of disease without the obligation to conduct a conformity assessment procedure, as long as safety standards and those for quality management are in place. Based on Article 5(5) of the EU Medical Device Regulation (MDR; 2017/745) [75], this exemption applies only for in-house use on a nonindustrial scale and if the needs of the targeted patient groups cannot be met through available and equivalent devices on the market [75,93]. Also covered is the in-house combination or modification of existing systems or devices [93,94]. For example, in Table 5**,** we have outlined 3 practical examples of AI systems, which have been developed in-house in a German hospital setting, each of them with a unique intended purpose, clinical indication, and target group. We highlight for each the technical approach used as well as the stakeholders included during development and potential prospective trial designs.

**Table 5:** Practical examples of AI applications developed in-house and their stakeholder integration. The AI use cases presented originate from the SmartHospital.NRW [95] research project, funded by the Federal State of North Rhine-Westphalia, Germany. The project is limited to research and development activities; therefore, the use cases are confined to the development stage. Clinical testing and product commercialization are explicitly beyond the project’s scope.

Use case	Automated discharge summary	AI^a^-powered voice assistant for bedside patient support	AI-supported prevention of adverse events
Intended purpose	Automates and optimizes the creation of discharge letters within hospital workflows to reduce clinician workload and improve communication regarding patient care.	Enables patients at the bedside to interact via natural speech, facilitating access to medication schedules, personal calendars, diary management, and support to overcome language barriers through oral translation and simplified language.	Focuses on early and reliable detection of nursing-relevant risks by enhancing existing risk models based on structured nursing assessments and integrating LLMs^b^ to analyze clinical progress notes and identify patient-specific risk factors.
Clinical indication	Addresses the challenge of time-intensive medical documentation, particularly discharge summaries following inpatient stays.	Designed for patients requiring accessible communication support, especially those experiencing language barriers, vision impairments, or limited mobility, while promoting autonomy without providing direct medical advice.	Designed to support systematic, early identification of nursing-related risks, including falls, pressure ulcers, and malnutrition, augmenting safety and enabling individualized care planning.
Target group	Primarily, hospital physicians with indirect patient benefits, such as improved continuity of care and efficient information transfer to general practitioners.	Hospitalized patients who require assistance in accessing information and communicating effectively.	Nursing staff responsible for patient care and hospitalized patients are actively involved in care processes.
Technical approach	Uses generative AI language models interfaced with hospital information systems to autonomously extract structured clinical data and generate contextually relevant text suggestions for documentation.	Uses on-premises LLMs within dedicated patient devices; enables localized processing of voice input streams independent of hospital system integration, thereby preserving data sovereignty.	Integrates structured clinical data, unstructured data derived from speech-to-text conversion of nursing assessments, and patient-reported outcomes to facilitate comprehensive risk detection.
Stakeholders included during development	Management Board, AI System Developer, AI-System Owner, IT Specialists, Clinical Experts, and Users.	Management Board, AI System Developer, IT Specialists, Clinical Experts, and Users.	Management Board, AI System Developer, AI-System Owner, IT Specialists, Clinical Experts, and Users.
Experience of development	Developed iteratively as a prototype, validated with real clinical data, while ensuring compliance with regulatory, privacy, and interoperability standards.	Followed an iterative development approach with thorough curation of informational content; faced technical challenges such as limited server access before full deployment of open-source models.	Development prioritized screening instruments to assess signs and symptoms of nursing care, optimization of AI risk detection models, and ensuring data privacy using pseudonymization and anonymization techniques.
Potential prospective trial designs	Cluster-randomized controlled trial at the ward level, comparing standard discharge processes versus AI-assisted summaries. Primary endpoints: clinician documentation time and report quality (as judged by independent review).	Patient-level crossover trial with and without AI voice assistant support. Main outcomes: patient autonomy, effectiveness of information access, and user satisfaction, controlling for intrapatient variability.	Pragmatic controlled trial in clinical wards comparing standard care with and without AI-based risk detection algorithms. Outcomes: incidence of adverse events (falls, pressure ulcers, and malnutrition), timeliness of risk identification, and changes in clinical workflow.

^a^AI: artificial intelligence.

^b^LLM: large language model.

In contrast to commercial deployments, in-house systems offer a distinctive opportunity for embedding participatory ethics, iterative design cycles, and real-world validation and feedback loops directly into the lifecycle of medical AI. This allows the creation of a highly customized solution that fits in location-specific clinical workflows and staff practices, which can be extended to multiple systems within the same platform and institution [76]. Moreover, a key advantage is the use of the hospital's own data; however, this requires a well-developed data infrastructure and processes for obtaining patient consent. Considerations include interoperability and data preparation, such as labeling (although label-free approaches are becoming more common), structuring, and collection (requirements also under the AI Regulation), in order to know which data can be used for a specific solution.

### Step 4: AI-System Testing, Validation, and Clinical Evaluation

Health care AI demands rigorous, multidimensional evaluation that must encompass not only technical performance, but also clinical integration, and verify safety, usability, ethical robustness, and regulatory compliance.

Independent assessment of device performance can be generated through statistically sound test plans, which generate information separate from the training data set [96]. Since validation in real-world settings is still a bottleneck [97], prospective, noninterventional silent trials [98,99] (where AI is tested within the clinical pathway in real time without affecting patients) can enhance transparency and facilitate informed deployment decisions. For large language models (LLMs) and, in particular, adaptive AI models that evolve over time, continuous validation frameworks are needed [100]. Recent studies have highlighted, substantial challenges to the reliability and safety of LLMs in health care persist, including hallucinations [101], metacognitive deficiencies [102], vulnerability to bias [103] and data-poisoning [104], and problems in integration in existing workflows [105], making single evaluation dimensions insufficient. Therefore, multidimensional methods could help to operationalize feasibility, score diagnostic accuracy or unsafe recommendations, and detect bias and usability issues. Examples are “QUEST” [106] to score outputs, or agentic-based simulations such as “CRAFT-MD” [107] for clinical workflow evaluation. Alignment with international AI standards (eg, ISO/IEC [International Organization for Standardization/ International Electrotechnical Commission] 42001:2023 [108], FG-AI4H [Focus Group on AI for Health] clinical evaluation framework [109]) further strengthens interoperability and safety.

Beyond objective data and algorithm quality, subjective feedback from users is essential [57,110]. Evaluations should capture how AI systems integrate into existing workflows and routines, their ease of use, and their perceived performance and interface design. Researchers highlighted several approaches for evaluation, such as through integrated feedback systems [110,111] or through organizational internalization by creating an “AI-QI”-unit responsible for quality improvement and assurance [87], interacting as a “glue” between different entities.

**Figure 4:**
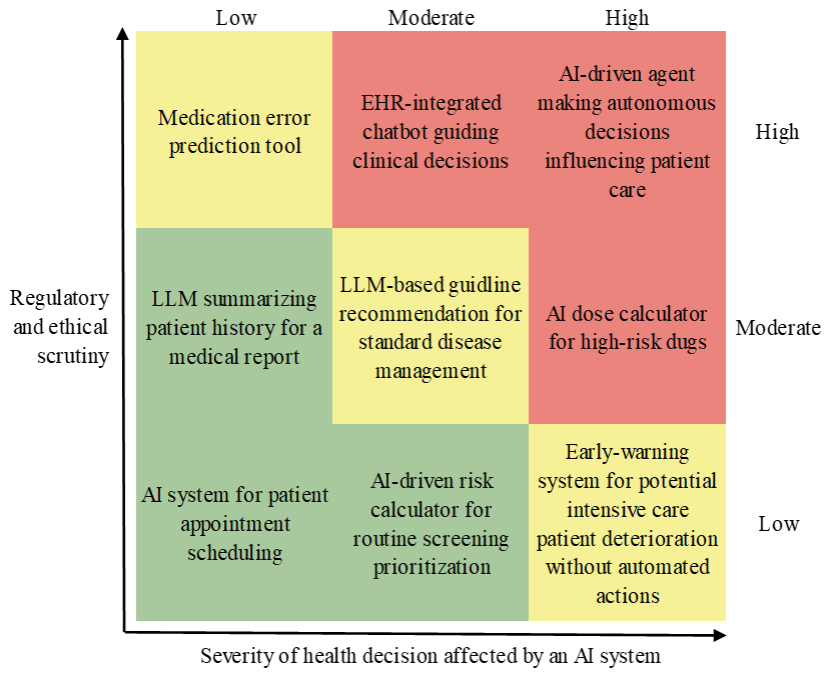
Risk-based tiering of safeguards. With a proportional approach to regulatory and ethical safeguards aligned with the severity of the health decisions affected by an AI system, this provides a useful link between risk classification (eg, under Medical Device Regulation or the EU AI Act) and the required level of human oversight, transparency, and stakeholder involvement. AI: artificial intelligence; EHR: electronic health record; EU: European Union; LLM: large language model.

Evaluation should follow a risk-tiered approach that links the level of regulatory and ethical scrutiny to the severity of the health decision involved (Figure 4). For instance, AI systems used for administrative optimization or appointment scheduling may require a lower level of risk mitigation, while those supporting diagnostic or therapeutic decisions demand significantly higher safeguards. This tiering can draw on the EU AI Act’s risk classes and MDR risk classifications, and should be developed in consensus with relevant stakeholders, including clinical risk management and regulatory specialists.

To ensure that the AI system is compatible with European values, ethics-based auditing frameworks like capAI, grounded in the EU AI Act, can guide risk identification in each phase of the AI lifecycle from an ethical point of view [112]. The integration of tools like the self-assessment list for trustworthy AI (ALTAI) [113], developed by the EU High-Level Expert Group on AI, into ethics-based auditing of AI systems can further support responsible usage of AI and foster user trust. Yet, ethical guidelines are just that: guidelines. They rarely or incompletely answer concrete ethical questions regarding the use of an AI system in a specific situation, such as the question of specific moral responsibility if mistakes of AI systems lead to patient harm. This is a highly discussed topic in ethics [114] and becomes even more severe in the context of black-box problems, eventually leading to moral responsibility gaps [115]. Other still unsolved ethical questions occur, for example, regarding data ownership in the context of the principle of beneficence (ie, promoting others’ benefit and preventing harm [116,117]) and informed consent [118] or anthropomorphization of AI [119]. Therefore, embedding ethical points of view into the whole life cycle of AI is necessary [120].

### Step 5: Development and Deployment of Training Approaches

The successful adoption of AI by hospital employees correlates with continuous development and training [88]. Although training is also a requirement of the EU AI Act [121], it is of note that only 24% of the health care institutions provide AI training programs and workshops [122]. This underscores a gap in education and certification, leaving clinicians without the necessary tools to harness the full potential of AI. However, there are various ways to support confidence in AI technologies among HCPs. For example, (1) by investing in comprehensive training programs that help to gain necessary skills [88] while also extending existing programs with AI literacy, or (2) by developing and provisioning resources and mechanisms to build and strengthen connections among peers and innovators to share their AI-related knowledge and experiences [80]. And more importantly, AI training should be a fixed part of any professional education and competency assessment, as well as included in further training (eg, through integration into Continual Medical Education programs) [123] to build confidence in its use among the next generation of HCP and achieve a symbiotic relationship between humans and AI [124].

In order to build AI literacy among HCP in a safe and controlled environment, training methods such as simulation-based modules [125,126] (ie, practice in realistic settings [125,127]), case-based exercises [128], and interactive workshops [129] can help to explore tools repeatedly without risking patient safety while facilitating experimental learning. Another method of providing HCPs with hands-on experience using AI tools in a controlled environment is to conduct a pilot phase, during which AI is tested by selected clinical users in a narrow area of practice, or shadow deployment, in which AI operates in shadow mode alongside clinicians in real time and is guided by predefined safety and workflow indicators [130]. This will also influence trust and adoption among users and foster psychological safety, since evidence from human-computer interaction research indicates that a positive attitude toward AI is not only a function of system transparency or explainability, but also depends on users’ self-efficacy, previous experience, and the perceived fairness and predictability of the system [131]. With regard to content, it is important to define responsibilities within the company regarding who will take ownership of training the users in basic competencies of AI literacy. The AI-System Owner and his or her team would be the best fit, as they combine the entire use case-relevant expertise through different perspectives, ranging from clinical experts to system developers.

**Figure 5:**
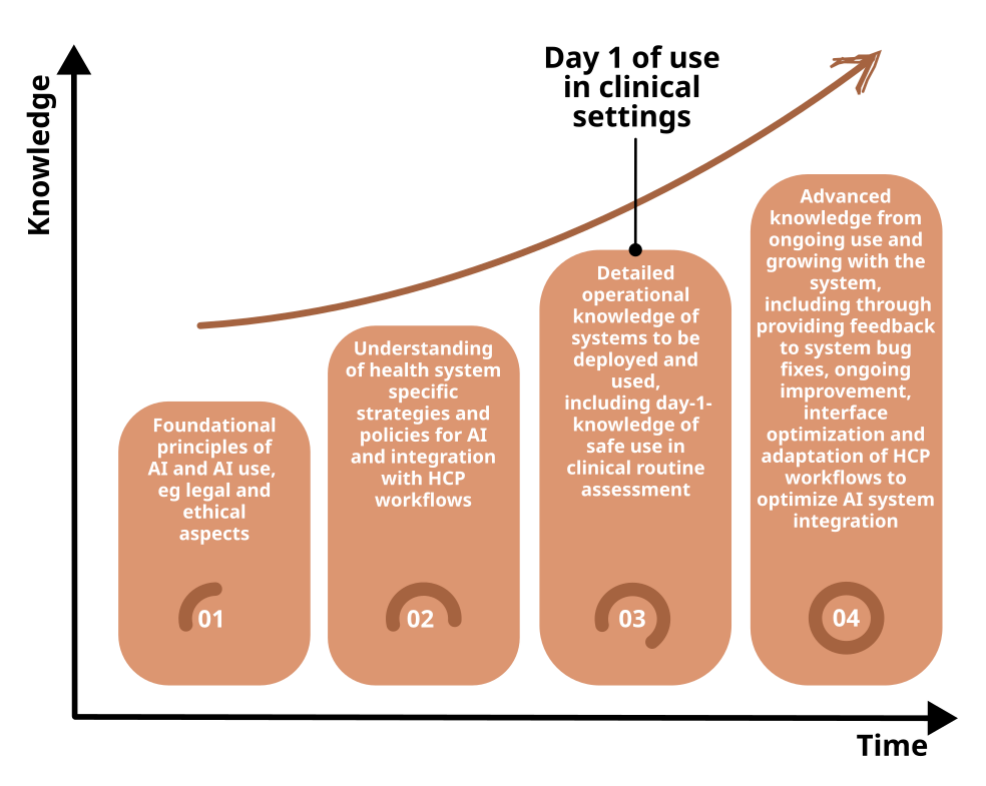
The learning curve of the health care professional (HCP) in the use of artificial intelligence (AI) systems in health care. After training in the basic AI principles and their use, as well as health care–specific guidelines for AI integration, on the first day of the system’s clinical use, the HCP should be trained in the operational knowledge of the system being deployed. The HCP will then develop their skills through experience in their use.

Training should foster understanding of AI systems and facilitate interaction and use of AI systems, and is relevant not just for direct users, but for all HCPs who will work alongside care systems influenced by AI (Figure 5) [15,66]. Key competencies are a basic understanding of when and how to use AI, knowledge about the use of the systems’ elements, the ability to make informed decisions based on a risk-benefit analysis, the awareness of legal and ethical considerations, and, to adapt to new tools and applications [123,132]. Components of health care AI training that are generic do not need to be developed de novo by the health institution. However, specific training directly related to the AI-system to be deployed will generally be required, and it is often necessary to provide ongoing training which takes account of the learning curve of the HCP in the use of the AI, emergent problems such as automation bias [133] and deskilling [134], and changes and further development of the AI-systems.

### Step 6: AI-System Deployment, Real-World Performance Monitoring, and Later Decommissioning

After model creation and testing, the goal is to place the system in real-world clinical settings to improve patient care and outcomes [135] according to the previously defined overall goals and device purpose. This needs transparency, and compliance with legal and ethical processes (user consent), as well as the completion of all steps required for the exemption to conduct a conformity assessment under in-house deployment (“MDR Article 5(5)”) or third-party approval (CE-mark). Therefore, looping in all stakeholders is needed to collaboratively address associated challenges. A key role is played by the management board and AI-System Owner to provide a clear external and internal communication that signals the prioritization of human well-being during the whole process, and users as multipliers to promote trust for broad widespread acceptance and use.

Involving all stakeholders also applies to monitoring and oversight of real-world performance, as it needs constant feedback from different perspectives to improve system performance and data-related processes. The goal of monitoring is to raise an alarm when unintended or special cases occur [87], which emphasizes the importance of finding solutions through collaboration and collective intelligence. The “AI-QI” unit described above could consolidate and strengthen the established stakeholder structure within the company long term. In addition, algorithmic audits can serve as a framework for continuously monitoring AI systems and understanding errors, how and why these adverse events occurred, while anticipating their potential consequences [136]. Real-world performance monitoring must adequately account for model drift (degradation of AI system performance over time) due to changes in external factors such as patient populations, data collection, or medical practice [137].

Running a “legacy system” usually means facing layers of technical debt, which slows down development and complicates maintenance, and leads to several risks, such as the technology becoming less reliable and decreasing in performance, or exposing systems to vulnerabilities such as cyberattacks. However, decommissioning can be an option to abstract and secure data in a newer system [138]. This process needs to be carried out by IT and regulatory specialists, as well as data scientists and quality management, in consultation with users, the management board, and employee representatives, and notified bodies where required.

## Special Considerations for Adaptive, “Agentic” and “off-the-Shelf” AI Systems

Some recent AI approaches are developed so that they learn and adapt from data and feedback from the real world, allowing them to change continuously without explicit interventions from the developer [139,140]. Ensuring such systems are safe, effective, and of high quality while being flexible requires a more interactive and participatory approach than traditional systems that follow static and predefined rules. This is especially true when self-learning systems are combined with agentic AI systems that are able to handle multilevel tasks, coordinate tools, centralize human communication, and basically act as health care teammates [26-29]. Autonomous AI systems and LLM-enabled clinical decision systems have already been approved in Europe [30,141,142]. As the approval and use increase, and as these systems continuously encounter new settings and tasks, it is essential to define clear boundaries, controlled environments with clinician oversight [27], ongoing auditing [26], and adequate training capacities for HCPs [27]. As broad models may be applied across multiple hospital departments and clinical contexts (eg, simultaneously used in an emergency department and psychiatry clinic) with dynamic or variable workflow integration, transparent communication, and iterative feedback across stakeholders (as presented in this paper) are also critical to ensure adaptability and to address the more complex ethical, legal, and social implications.

For off-the-shelf AI systems provided by external companies, the interaction between stakeholders should be focused on integration, compliance, and validation to meet operational and regulatory needs. These systems may limit the level of innovation achievable (no bottom-up activism from internal users and developers to continually contribute improvements and features that better meet unique requirements) and may lead to trust issues due to less transparency in the handling of data and underlying algorithms [14], requiring proactive communication and change management. Responsibilities for monitoring and model updating, especially with proprietary algorithms, become more complex and need to be clarified between external collaborators and internal stakeholders [87]. Platforms for delivering off-the-shelf AI systems now allow the co-hosting of in-house developed AI models, alongside the CE-marked models, enabling both approaches to coexist, and making clear the need and possibilities for the co-design, embedding, and co-implementation of commercial and in-house approaches [143].

## Discussion

Studies show a persistent gap between research and clinical implementation [144,145], with medical AI adoption still very slow [144,146] and limited to a few use cases [147]. Reasons include the difficulty of aligning diverse stakeholder perspectives within complex health care systems, the rigidity of regulatory frameworks, and the limited consideration of design approaches of work and organizational psychology [148]. As a result, achieving both technological effectiveness, in the sense of medical accuracy and system performance, and user acceptance among HCP and patients is often perceived as conflicting goals.

A balance is therefore needed between ensuring safety and enabling innovation [149]. EURAID finds this “sweet spot,” accelerating digital transformation in a human-centric way. Unlike existing frameworks, which focus narrowly on user perspectives [80,150,151], isolated implementation aspects [150,152-155] (such as evaluation, safety, or ethics), serve as decision support tool for choosing the most fitting available AI solution [156], or have a limited clinical scope [157-160], EURAID explicitly maps all key stakeholders across the AI development life cycle, clarifies their roles and key aspects they can address (Table 4) in co-creating, guiding, and governing “in-house” AI development and deployment. It also details stakeholder roles in real use cases, and methods for achieving iterative consensus at each development stage across disciplines that reflect shared goals in alignment with European values, and strengthening the understanding of training methods, content, and key competencies.

However, EURAID has some limitations. The resources or specialized staff needed for iterative development and testing are more limited in smaller hospitals, necessitating concentrating multiple roles on fewer people, which can lead to a shortage of expertise, but, on the other hand, may also speed up processes. Although our approach can likely better address creative problem-solving, traditional, rigid, and hierarchical structures common in health care may hinder stakeholder selection based on their contributions and expertise rather than their positions and level of seniority. Although “in-house” AI devices may not require CE marking, they are not exempt from regulation and have legal liability implications. Health institutions must comply with a number of obligations that may discourage them from doing it at all, which slows down both innovation and digitalization. A practical solution is to designate key staff for legal or ethical liaison roles or establish a multidisciplinary AI advisory board and data governance council within the institution to ensure compliance and continuity.

### Conclusions

EURAID is a pragmatic, solution-oriented framework, compatible with European values and regulations, and ensures that barriers to “in-house” AI development and implementation in hospitals are acknowledged early and resolved through collaborative problem-solving. The underlying principle is that the likely future of medicine, driven by integrated, localized, and adaptive AI technologies, will need all critical stakeholders (which we portray individually in this paper) adequately represented, and their various perspectives embedded in the co-design, procurement, implementation, and oversight of AI systems, ensuring that digital transformation in health care truly benefits the people who will use them every day. Additionally, as AI systems used vary by type and clinical setting, we propose a risk-tiered approach that provides a useful link between risk classification and the required level of human oversight, transparency, and stakeholder involvement.

To translate EURAID into action, hospitals should begin by conducting internal readiness assessments, establishing cross-functional AI governance structures, and defining clear, role-specific responsibilities for ethical, legal, technical, and clinical oversight. Regulators and professional bodies should, in parallel, create structures that connect local innovation with next-generation European legislation, for governance that is as intelligent as the technology built.

## Abbreviations

AI: artificial intelligence
CE: Conformité Européenne
EU: European Union
EURAID: European Responsible AI Development
FG-AI4H: Focus Group on AI for Health
HCP: health care professional
ISO/IEC: International Organization for Standardization/ International Electrotechnical Commission
LLM: large language model
MDR: Medical Device Regulation
NHS: National Health Service
SMART: specific, measurable, attainable, relevant, and time-bound
TEU: Treaty on European Union
XAI: explainable AI
